# Public health approaches and policy changes after the inclusion of gaming disorder in ICD-11: global needs

**DOI:** 10.1192/bji.2021.57

**Published:** 2022-08

**Authors:** Jiang Long, Roshan Bhad, Marc N. Potenza, Laura Orsolini, Vicky Phan, Mitika Kanabar, Sophia Achab

**Affiliations:** 1 Psychiatrist/Researcher, Shanghai Mental Health Center, Shanghai Jiao Tong University School of Medicine, Shanghai, China; 2 International Society of Addiction Medicine (ISAM)-NExT (New Professionals Exploration, Training & Education Committee); 3 National Drug Dependence Treatment Centre, All India Institute of Medical Sciences (AIIMS), New Delhi, India; 4 Department of Psychiatry and Child Study Center, Yale School of Medicine, New Haven, CT, USA; 5 Unit of Clinical Psychiatry, Department of Neurosciences/DIMSC, School of Medicine and Surgery, Polytechnic University of Marche, Ancona, Italy; 6 Turning Point, Eastern Health, Melbourne, Australia; 7 Southern California Permanente Medical Group, California, USA; 8 Psychological and Sociological Research Unit, Faculty of Medicine, University of Geneva, Switzerland. Email Sophia.achab@unige.ch

**Keywords:** Education and training, gaming disorder, public health and policy making, ICD-11, management

## Abstract

The World Health Organization (WHO) has added gaming disorder to ICD-11 as a clinical condition associated with distress or interference with personal functioning. This inclusion leads to clinical and public health benefits, such as harmonising terminology, offering clinical landmarks and improving monitoring capabilities and data comparability. Training health professionals to identify and manage gaming disorder is a key challenge for countries. In the present paper we compiled opinions from different countries around the globe on their state of preparedness and needs to tackle this issue. The global views on the topic feed arguments for developing an evidence-based and cross-cultural training tool for gaming disorder management by health professionals.

## The inclusion of gaming disorder in ICD-11

Video gaming, especially internet gaming, is a popular pastime of youth and adults worldwide. However, a significant minority of gamers experience functional, psychological, social and physical impairment as a result of excessive gaming.^1^ This has raised public health concerns globally and calls for diagnostic criteria for gaming disorder.^2^ In ICD-11 gaming disorder has been defined by the World Health Organization (WHO) as a clinical condition associated with distress or interference with personal functioning.^3^ It is included in a new disease category of ‘disorders due to addictive behaviours’, which was adopted at the World Health Assembly in May 2019 and will come into effect in January 2022.

The inclusion of gaming disorder has been debated among researchers, clinical practitioners and public health specialists.^4–6^ From clinical and public health perspectives, we, as committee members of the International Society of Addiction Medicine (ISAM) and researchers/clinical practitioners in addiction, believe that the inclusion is an essential response to marked distress and functional impairment in people experiencing gaming disorder, and the associated demand for intervention and treatment. As WHO member states will use ICD-11 for morbidity and mortality statistics, reimbursement systems and automated decisions on support in healthcare, the inclusion will support progress in clinical and public health approaches to gaming disorder worldwide. Present global healthcare challenges associated with gaming disorder include: (a) increased care needs among affected people and their relatives; (b) the lack of a gold standard for screening and assessment for the disorder; (c) the lack of broadly accepted criteria to diagnose and treat patients; and (d) health professionals’ preparedness to accurately identify and manage gaming disorder.

The inclusion of gaming disorder leads to public health benefits in multiple ways. First, the recognition of gaming disorder as a disease should facilitate research by providing a standard definition and criteria for international researchers. Second, the inclusion may improve specialised clinical services in countries through improved awareness of diagnostic criteria and symptom domains to guide management. Appropriate clinical management and treatment may be further developed and delivered with accumulating evidence from clinical practice, as research and experience in managing gaming disorder expand and are streamlined. Recognition of this diagnosis means that in some jurisdictions, persons experiencing the disorder may be entitled to subsidies and/or reimbursement for treatment of their disorder through healthcare subsidies or insurance. Third, the inclusion of gaming disorder may enable capacity building among health professionals (e.g. specialised training for professionals) and promote communication among service providers across countries, which can foster better clinical understanding and management of the disorder.

## International responses to gaming disorder

Owing to various differences (e.g. cultural, economic, administrative, position of local gaming industries) between countries, responses to gaming disorder at national levels differ. As it is crucial to map countries’ preparedness and needs for capacity building among healthcare professionals to address gaming disorder, we therefore collected international cases from countries where we are located and practising as examples to showcase public health approaches and policy efforts to manage gaming disorder, following its approval for inclusion in ICD-11. Global views from different countries and continents are presented below in alphabetical order.

### Australia (national case supplied by Vicky Phan)

The prevalence of gaming disorder in Australia is unknown and confidence in the concept of gaming disorder, and its assessment and management, has not been ascertained following its inclusion in ICD-11. Presently there is no national action strategy to address gaming disorder in Australia and professional colleges representing addiction medicine specialists, the Royal Australia and New Zealand College of Psychiatrists and Royal Australasian College of Physicians have not developed position statements or clinical practice guidelines relating to gaming disorder. It is likely that capacity building would be needed to support Australian health professionals to diagnose and manage persons affected.

### China (national case supplied by Jiang Long)

The National Health Committee (NHC) of China and other governmental agencies welcome the inclusion. In fact, prior to the inclusion the authorities had developed a series of regulations on online game services (e.g. restriction on gaming time for minors), which aim to limit access to online games for minors in response to public health concern caused by excessive gaming. The inclusion has greatly facilitated technical guidance on gaming disorder. In 2019, an NHC consensus^7^ on the prevention and treatment of gaming disorder presented current knowledge on the disorder and encouraged further study into this area. In 2020, the committee published guidelines on the diagnosis and treatment of mental disorders^8^ which include a section on gaming disorder and its clinical management. However, with regard to capacity building among professionals in managing gaming disorder, there are presently no substantial efforts at a national level in China, although several leading medical institutes in big cities (e.g. Shanghai, Changsha) have organised training on medical management of gaming disorder on a small scale. As government documents state, capacity building among health professionals in the management of gaming disorder is a priority area in the future for China.

### Italy (national case supplied by Laura Orsolini)

In Italy, initiatives targeting mental health professionals, physicians and teachers have been rapidly implemented to limit or reduce the risks and physical/mental health consequences of gaming disorder, particularly in youth. Primarily these initiatives have focused on education and early identification, with few interventions/actions implemented to build mental health professionals’ capacity in gaming disorder management. Italy's National Prevention Plan 2020–2025^9^ seeks to implement preventive activities and strategies to screen and identify individuals at risk of developing gaming disorder. The plan also stipulates the need to implement educational activities regarding gaming disorder to raise awareness. Furthermore, an agreement between the Italian Ministry of Education, University and Research and the Italian Ministry of Health was signed in February 2019, acknowledging an emergent need to act on gaming disorder, including promoting educational activities on the disorder. However, there is still a lack of specific initiatives seeking to develop the health professional workforce in the field of gaming disorder. Therefore, further educational and supporting activities should be implemented at university level considering this new emerging issue.

### India **(**national case supplied by Roshan Bhad)

Epidemiological studies on gaming disorder in India are limited. There are no nationwide data available on the problem. The inclusion of gaming disorder in ICD-11 is likely to improve identification, generate epidemiological data and encourage treatment of the problem in India. However, it is important to distinguish between normal gaming behaviour and gaming disorder and clarity will be needed regarding the term ‘hazardous gaming’, also introduced in ICD-11.^10^ Currently there are few dedicated clinics for treatment of behavioural addictions in India; hence, capacity building and allocation of resources and training will be key to identifying and addressing gaming disorder in India. There are no specific initiatives to develop a workforce in the field of gaming disorder in India, which is a major challenge to addressing this emerging problem in the country.

### Switzerland (national case supplied by Sophia Achab)

Excessive gaming has been recognised as an area of concern by the Swiss Federal Council since 2012, and public health actions have been included in national strategic plans^11,12^. There is no defined national plan aiming at preparing healthcare professionals to address gaming disorder, and capacity building has been identified as a priority area of need for addiction medicine professionals in a national survey conducted in 2017. A first national step has been the development under the auspices of the Ministry of Health of an intervention guide for professionals counselling parents about screen use, including gaming, released in 2020.^13^ Nonetheless, training for treating gaming disorder has been organised locally for the past decade by leading academic institutes in big urban areas. Pressing short-term needs in Switzerland are to train in a harmonised way professionals following evidence-based standards (ICD-11 and the recommendations of global experts).

### USA **(**national case supplied by Marc Potenza & Mitika Kanabar)

In the USA, despite the prevalence of gaming disorder and need for services, there is no national consensus or guideline for the diagnosis and treatment of the disorder. Although the government has shown interest in researching and understanding the impact of screen media activity that includes gaming and gaming problems (e.g. https://grants.nih.gov/grants/guide/rfa-files/RFA-HD-22-009.html), as have private foundations such as Children and Screens, a unified, overarching governmental approach is arguably absent. There is arguably no specifically defined federal strategic plan to address gaming disorder. Nonetheless, there exist efforts at state and national level to increase awareness of gaming disorder and provide help for people with such concerns. At a state level, many agencies (e.g. the Departments of Mental Health and Addiction Services) have provided training to educate healthcare providers about gaming disorder. Often, agencies that focus on treating people with gambling problems (e.g. state affiliates of the National Council on Problem Gambling) are in advantageous positions, given their experience in treating people with this behavioural addiction and the convergence of gaming and gambling behaviours.

## Conclusions

The inclusion of gaming disorder in ICD-11 brings positive implications from clinical and public health perspectives. Following our review of international responses to the inclusion of gaming disorder, a common need for capacity building in the management of gaming disorder has been identified across countries. Although there is a pressing need for evidence-based training of healthcare providers in screening, assessment and/or treatment of gaming disorder, reliable training tools are globally scarce at present, despite some advances made.^14^ Ideally, such training tools (c.f. the WHO Mental Health Gap (mhGap) Action Programme intervention guide on mental, neurological and substance use disorders)^15^ may be developed by international organisations (e.g. WHO) or international academic associations that can mobilise international resources more easily, as they could be best informed by contemporary global evidence and extensive feedback and recommendations from international experts. Collective international efforts are imperative to develop high-quality training tools that can be adapted and utilised in various cultural settings for healthcare providers. Moreover, the cost-effectiveness of scaling up care interventions should apply to gaming disorder as are such interventions deemed as a crucial consideration in the management of mental health conditions.^16^ Taking the example from the WHO mhGap programme,^17^ the lack of specialised health professionals for gaming disorder's identification and management in many jurisdictions and geographical areas could be compensated by disseminating affordable training materials for non-specialised health professionals.

## Data availability

Data availability is not applicable to this article as no new data were created or analysed in this study.
